# Is ferroptosis involved in ROS-induced testicular lesions in a varicocele rat model?

**DOI:** 10.1186/s12610-021-00125-9

**Published:** 2021-04-01

**Authors:** Erfaneh Shaygannia, Mohammad H. Nasr-Esfahani, Fattah Sotoodehnejadnematalahi, Kazem Parivar

**Affiliations:** 1 Department of Biology, School of Basic Sciences, Science and Research Branch, Islamic Azad University, Tehran, Iran; 2 Department of Animal Biotechnology, Reproductive Biomedicine Research Center, Royan Institute for Biotechnology, ACECR, Isfahan, Iran

**Keywords:** Varicocele, Reactive oxygen species, Ferroptosis, Alpha-lipoic acid, Varicocèle, Espèces réactives de l’oxygène, Ferroptose, Acide alpha-lipoïque

## Abstract

**Background:**

Ferroptosis is an iron-dependent cell death that is distinct from apoptosis. Based on excessive amounts of iron and reactive oxygen species in varicocele (VCL) rats, we hypothesize that ferroptosis might be involved in VCL. In addition, since alpha-lipoic acid (ALA) was shown to have both antioxidant and anti-ferroptotic activity we assessed in the present work the status of ferroptosis in our varicocele model and the protective effect of ALA. To this end, 70 male Wistar rats were divided into 7 groups: control, sham and varicocele groups which were initially sacrificed 2 months after the operation to verify the induction of varicocele. A second batch of the same 3 groups were sacrificed 4 months after varicocele induction to evaluate the effect of ALA supplementation. The parameters measured were chromatin integrity (aniline blue and acridine orange staining), lipid peroxidation (BODIPY staining), testicular morphometry and iron content. In addition, redox (GSH and NADPH) and ferroptosis (Nrf2, Slc7a11, P53 and p-Jnk) markers were evaluated at 2 and 4 months post-operation.

**Result:**

The alteration of the spermatic parameters made it possible to verify the induction of the varicocele. Iron accumulated well in the testicles during varicocele and decreased significantly following ALA treatment. Ferroptotic molecular markers at the mRNA and protein levels were not significantly altered. ALA supplementation did not alter NADPH values, but increased GSH levels.

**Conclusion:**

Despite the increased accumulation of iron in the testes 2 and 4 months after surgical induction of varicocele, molecular evidence did not demonstrate the involvement of ferroptosis. This could be explained by the mosaic nature of the varicocele affecting some seminiferous tubules and not others which could mask variations in molecular markers. In parallel, our study confirms that ALA stimulates the NRF2 pathway.

## Résumé

**Contexte:**

La ferroptose est une mort cellulaire dépendante du fer qui est distincte de l’apoptose. Sur la base de quantités excessives de fer et d’espèces réactives de l’oxygène chez les rats varicocèles (VCL), nous avons fait l’hypothèse que la ferroptose pourrait être impliquée dans la VCL. Comme l’acide alpha-lipoïque (ALA) s’est avéré avoir des activités antioxydante et anti-ferroptotique nous avons dans ce travail testé le statut ferroptose dans notre modèle de rats VCL et évalué l’effet protecteur de ALA. Dans ce but, 70 rats mâles Wistar ont été divisés en plusieurs groupes: témoin, fictif et groupes varicocèle qui ont été initialement sacrifiés deux mois après l’opération pour vérifier l’induction de la varicocèle. Un deuxième lot des 3 mêmes groupes a été sacrifié 4 mois après l’induction de la varicocèle pour évaluer l’effet de la supplémentation en ALA. Les paramètres mesurés étaient l’intégrité de la chromatine (via les tests au bleu d’aniline et à l’orange acridine), la peroxydation lipidique (via le test BODIPY), la morphométrie testiculaire et la teneur en fer. De plus, les marqueurs redox (GSH et NADPH) et ferroptotique (Nrf2, Slc7a11, P53 et p-Jnk) ont été évalués 2 et 4 mois après l’opération.

**Résultats:**

L’altération des paramètres spermatiques a permis de vérifier l’induction de la VCL. Le fer s’est bien accumulé dans les testicules pendant la VCL et a diminué de manière significative après le traitement ALA. Les marqueurs moléculaires ferroptotiques n’ont pas été modifiés de manière significative que ce soit en quantité d’ARNm et de protéines. La supplémentation en ALA n’a pas modifié la teneur en NADPH, mais a augmenté les niveaux de GSH.

**Conclusions:**

Malgré l’accumulation accrue de fer dans les testicules 2 et 4 mois après l’induction chirurgicale de la VCL, les investigations au niveau moléculaire n’ont pas clairement démontré l’implication de la ferroptose. Cela pourrait s’expliquer par la nature mosaïque de la VCL qui affecte certains tubules séminifères et pas d’autres, ce qui pourrait atténuer les évaluations réalisées sur organe entier. En parallèle, notre étude confirme que l’ALA stimule la voie NRF2 stimulant la réponse anti-oxydante.

## Introduction

Programmed cell death or apoptosis plays a fundamental role in development, tissue homeostasis and various diseases. More recently, another process of programmed cell death, genetically and biochemically distinct from apoptosis, has been described [1, 2]. This new cell death process called ferroptosis is iron-dependent and is further characterized by an accumulation of iron-dependent lipid peroxides [1, 2]. In contrast to apoptotic cells, ferroptotic cells have soft plasma membranes, high-density mitochondria with diminished or disappeared crista and a normal nucleus with intact chromatin [3].

One of the biochemical characteristics of a ferroptotic cell is the reduction of glutathione (GSH) and nicotinamide adenine dinucleotide phosphate (NADPH), the latter being necessary for the recycling of the former. In addition, ferroptotic cells have a distinct gene expression profile [4]. One of the pathways involved in ferroptosis is the cystine (Cys2)/Glutamate Antiporter System x_c_^−^. This antiporter imports Cys2 into cells and exports glutamate from them. Inside the cells, Cys2 is reduced to cysteine (Cys) and becomes GSH after the addition of glutamate and glycine [1, 3]. Under normal conditions, GSH is used to control lipid peroxidation. In ferroptosis the iron-induced excessive lipid peroxidation via a process called the “Fenton reaction”, leads to GSH and NADPH depletion. It is therefore assumed that by inhibiting the Fenton reaction via iron chelation it is possible to limit ferroptosis [3].

The signaling pathways involved in ferroptosis are not fully understood, but it is proposed that the mitogen-activated protein kinase (MAPK) pathway plays an important role in this process. In addition, it has been revealed that P53 may also play a role in the ferroptosis pathway because it regulates the transcription of the *Slc7a11* gene, a subunit of the x_c_^−^ system [5, 6]. In addition, increased expression of Nrf2 (nuclear factor erythroid 2-related factor 2), a key player in antioxidant defense, stimulates the transcription of antioxidants such as Slc7a11 and Gpx-4 (Glutathione peroxidase 4) which have shown anti-ferroptotic activity [7, 8].

In varicocele (VCL) situations Gholirad et al. (2016) showed that due to reduced blood flow and increased testicular temperature, excessive amounts of iron are deposited in the testicles of varicocele rats [9]. Therefore, based on this observation, we hypothesize that ferroptosis could be activated. To test this hypothesis, we measured a set of markers of ferroptosis in the testes of a model rat in which VCL was induced. In addition, we evaluated the protective effect of alpha lipoic acid (ALA) on the level of ferroptosis, having previously shown that ALA could improve the structural and functional sperm parameters in this VCL rat model [10].

## Materials & methods

Seventy male Wistar rats were obtained from the breeding station of the Royan Institute of Biotechnology (Isfahan, Iran) and animal care was carried out as defined by the animal ethics committee of the ROYAN Institute. All rats were kept at a controlled temperature of 21 °C (±2%), a humidity level of 65% (±5%), a 12-h light/12-h dark cycle and ad libitum access to food and water.

### Study design

**Fig. 1:**
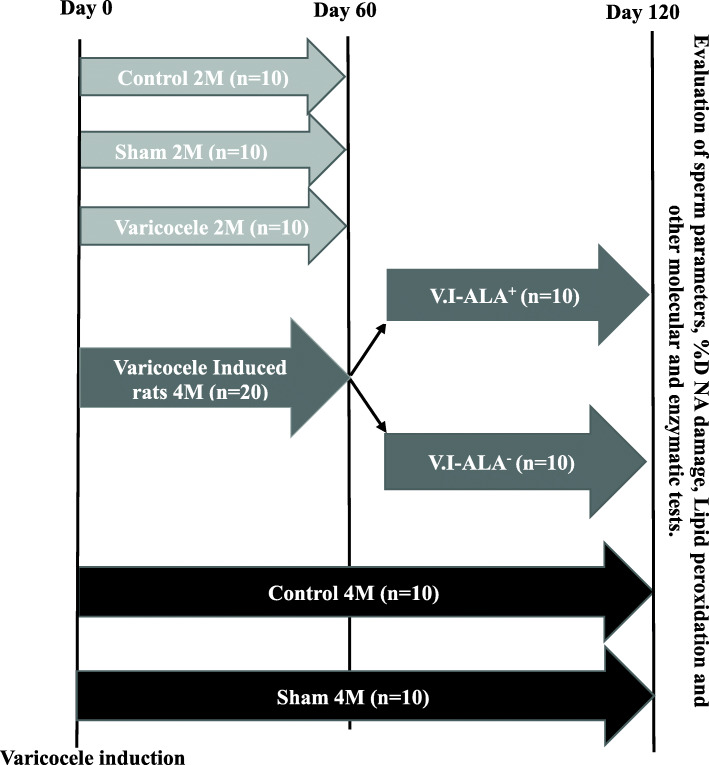
Study design. Legend: 70 male rats were used in this study, 10 in each group. Control, sham 2 M, varicocele 2 M, V.I-ALA^+^ (varicocele (V.I) rats treated with alpha lipoic acid (ALA) for 2 to 4 months post induction of varicocele), V.I-ALA^−^ (varicocele rats treated with water as solvent of ALA for 2 to 4 months post induction of varicocele), Sham 4 M and control 4 M. At day 60 (2 M) and day 120 (4 M) sperm parameters, %DNA damage, lipid peroxidation and other molecular and enzymatic tests were evaluated. ALA: Alpha lipoic acid, M: month post-surgery or varicocele induction (V.I). n: number of rats. +/^_^ presence or absence of ALA

Briefly, seventy male rats weighing approximately 180–210 g (8–9 weeks) were randomly divided into 7 groups (10 rats/group). Twenty rats were assigned to the control group. Twenty rats were assigned to the sham panel and the remaining 30 rats underwent surgery to induce varicocele by partial blockage of the left renal testis vein [11, 12]. Ten rats from each group (2 M control, 2 M sham and 2 M VCL) were sacrificed 2 months post-surgery for verification of varicocele induction. The remaining 10 rats from the control (4 M control) and sham (4 M sham) groups were kept for up to 4 months after surgery. Half of the 20 animals in the VCL group were gavaged daily with R-ALA (300 mg/kg of body weight, RAHA pharmaceutical company, Isfahan, Iran) dissolved in water (V.I-ALA^+^) while the remaining 10 rats were simply gavaged with water (V.I-ALA^−^). Rats at 4 months post-surgery were sacrificed to assess semen parameters (Fig. 1). In order to avoid inter-observer variations, all the assessment were carried out by a single well-trained individual.

### Biometric and histological analysis

After sacrifice, the left testis and epididymis were dissected and the volume of the testis was determined by the volumetric method using phosphate buffer saline (PBS), which is calculated by the difference between first and final volume. For the histological study, part of the testicular tissue was cut and fixed in bunion solution [13]. These samples were then dehydrated, fixed in paraffin and treated in 4–5 μm sections on glass slides maintained at 4 °C until stained with hematoxilin and eosin [13]. In order to evaluate the mitotic index at least 200 seminiferous tubules were evaluated per slide (200x magnification). The ratio between round spermatids and primary spermatocytes was calculated for each sample. The percentage of spermatogenesis was evaluated by observing 200 seminiferous tubules on each slide (200x magnification). The observation of spermatozoa was evaluated as a positive spermatogenesis index while the absence of spermatozoa was considered a negative spermatogenesis index. The result was expressed as a percentage of the positive spermatogenesis index. Johnsen’s score was evaluated according to the criteria originally described by Johnsen in 1970 [14].

### Sperm collection

Sperm were collected after incubation of diced cauda epididymis in 2 ml VitaSperm medium (Inoclon, Tehran, Iran) at 37 °C for 30 min. Sperm concentration and epididymal sperm motility were evaluated using a sperm counting chamber (Sperm Meter; Sperm Processor, Aurangabad, India) and an optical microscope (OPTIKA B-383LD2, Ponteranica, Italy). Sperm morphology was evaluated after staining with eosin-nigrosin (Merck, Germany). At least 100 cells were evaluated for each sample [15].

### Assessment of sperm DNA fragmentation, chromatin maturation and lipid peroxidation

Sperm DNA fragmentation was assessed by acridine orange (AO) staining. Briefly, the air-dried smears were fixed with Carnoy fixing solution (methanol/acetic acid (3:1)) at 4 °C for 2 h. Consequently, the slides were incubated with AO (final concentration: 0.1% in citrate buffer pH = 2.5, Merck, Germany) for 90 min. Samples were then stabilized in PBS. A fluorescence microscope (BX51 TRF, Olympus, Tokyo, Japan) was used to evaluate the percentage of AO positive sperm. While sperm chromatin condensation was monitored using aniline blue (AB) staining (final concentration: 5% AB in 4% acetic acid, pH = 3.5, Sigma, USA) as described previously [10]. whereas an optical microscope was used to evaluate the percentage of sperm AB positive. For each test, at least 200–300 sperm were counted on each slide.

Sperm lipid peroxidation (LPO) was evaluated using 5 mM of C11 BODIPY 581/591 dye (D3861, Molecular Probes, Junction City, OR, USA) at 37 °C for 30 min. The percentage of LPO for each sample was assessed using a FACSCalibur (Becton Dickinson, San Jose, CA, USA) Flow Cytometer as already described [16].

### Assessment of testicular Fe^3+^ distribution

The Perl’s Prussian blue staining method was applied to evaluate the distribution of Fe^3+^ [17]. Briefly, 4 μm sections of testes were dewaxed and dehydrated and exposed for 20 min to a mixture of potassium ferrocyanide (2%) and hydrochloric acid (2%) in a ratio of 1:1(v/v). The sections were then washed with water for 2 min and then fast red was used for 5 min at room temperature. Finally, the sections were successively washed with water and treated with alcohol and xylene. The images were obtained under the same conditions for all groups and the Fe^3+^ distribution was evaluated using the Image J software (version 1.42, National Institutes of Health, Bethesda, MD, United States) [17]. We used the surface to define the measurement window for the purpose of analyzing the quantity of Fe^3+^. We have also selected the pixel intensity unit for scaling the concentration values of Fe^3+^.All products necessary for this test were purchased from Merck (Germany).

### Relative gene expression analyses

To assess the level of expression of chosen genes, 50 mg of frozen testicular tissue was cut and chopped for homogenization. Extraction of total RNA was performed using the Trizol reagent extraction method (Sigma, USA) [18]. RNA quality was evaluated by measuring absorbance at 260 nm with a Nanodrop spectrophotometer (Thermo Fisher Scientific, Wilmington, Delaware USA). The samples were then treated with Dnase1 (Fermentas, Burlington, Canada) to avoid DNA contamination. Complementary DNA (cDNA) synthesis was performed using the Takara Revert Aid First Strand cDNA Synthesis Kit (Takara, Otsu, Japan) on 2 μg of RNA according to the manufacturer’s instructions. RT-PCR was performed using the ABI thermal cycling device according to the manufacturer’s instructions (Thermo Fisher Scientific, Foster City, CA, USA). Specific primers (shown in Table 1) were designed using Oligo 7 (Molecular Biology Insights, Inc., Cascade, CO, USA) and Beacon designer 7.5 software (Premier Biosoft, San Francisco, CA, USA). The final volume of each PCR reaction was 10 μl, containing 1 μl of each forward and reverse primers (5 pmol/μl each), 50 ng cDNA, 5 μl SYBR® green and 0.2 μl ROX. The reaction was carried out in triplicate. The qRT - PCR program was as follow: 15 s at 95 °C and 10s for the target genes, then 30 s at 72 °C. All data collected by qRT-PCR were normalized to the expression data of the GAPDH mRNA as a housekeeping gene. Relative gene expression was reported as 2^-ΔΔCT^ [18].

**Table 1:** Primers used in the study

Genes	Forward Primer	Reverse Primer	Annealing Temp (°C)	Product Size (bp)
***P53***	CCGACTATACCACTACCATAC	CACAAACACGAACCTCAAAC	64	147
***Nrf2***	GCCATTAGTCAGTCGCTCC	GTGCCTTCAGTGTGCTTCT	61	98
***Slc7a11***	TGTCTCCAGGTTATTCTATTTG	CCAGAGAAGAGCATTATCATG	54	138

*bp* base pair, *Nrf2* Nuclear factor erythroid 2–related factor, *Temp* Temperature, *°C* Degree Celsius

### Relative protein expression analyses

Protein extraction was performed on frozen testicular tissue by the Trizol precipitation method. Protein concentrations were evaluated using the Bradford’s test (Bio-Rad; USA). Equal amounts of each protein (30 μg) were loaded onto 12% SDS polyacrylamide gels (PAGE) which were then transferred to PVDF membrane (Bio-Rad; USA). Blocking solutions were prepared separately for each antibody. Nrf2 (1/500, Anti-rabbit, Abcam, USA), Slc7a11 (1/500, Anti-rabbit, Abcam, USA), Gpx-4(1/1000, Anti-rabbit, Abcam, USA), p-Jnk (1/1000, Anti-mouse, Abcam, USA) and β-Actin (1/500, Anti-rabbit, Santacruz, USA) were used as primary antibodies. Polyclonal IgG-HRP goat anti-mouse (1/5000, Dako, Denmark) and IgG-HRP goat anti-rabbit (1/16000, Santacruz, USA) were then used as secondary antibodies. Detection was performed using the GE Amersham ECL and Western Blotting kits (Amersham; GE Health care) and autoradiography [19]. Signal quantification was performed using Image J software.

### Assessment of testicular GSH content and NADPH content

GSH content was evaluated according to the Ellman method [20] with some modifications. Briefly, 50 ng of frozen testicular tissue was cut and homogenized using a soft homogenizer (Navand salamat, Uromiah, Iran). After addition of 5% TCA (Tricholoro acetic acid) and centrifugation for 5 min the supernatant were recovered. To 500 μl aliquot, 2 ml phosphate solution and 500 μl DTNB (5, 5′-dithio-bis-[2-nitrobenzoic acid]) were added. The GSH content was measured at 412 nm by spectrophotometry (Eppendorf, England). To specify the exact GSH content, a standard graph was established by preparing 5 to 50 mg/ml of pure glutathione in distilled water.

In order to evaluate the NADPH content, a NADP/NADPH assay kit (ab65349, Abcam, Cambridge, MA, USA) was used and the protocol was performed according to the manufacturer’s instructions.

### Statistical analysis

All data collected in this study were analyzed using SPSS (SPSS 19, SPSS, Chicago, IL, USA), and figures and tables were prepared using Graph Pad Prism 6. The one-way ANOVA with Tukey HSD for Post-Hoc analysis to estimate differences between means was used to compare means between groups (more than 2 groups) for each parameter. The independent sample T-test was used to compare data between 2 groups (control and 2 months varicocele). The data are presented as mean ± SEM. *P* < 0.05 indicates that a significant effect was observed. It should be noted that the objective of this study was not to make comparisons over time. The choice of the 2-month time-point was only intended to justify the establishment of varicocele as described earlier.

## Results

### Sperm parameters

**Fig. 2:**
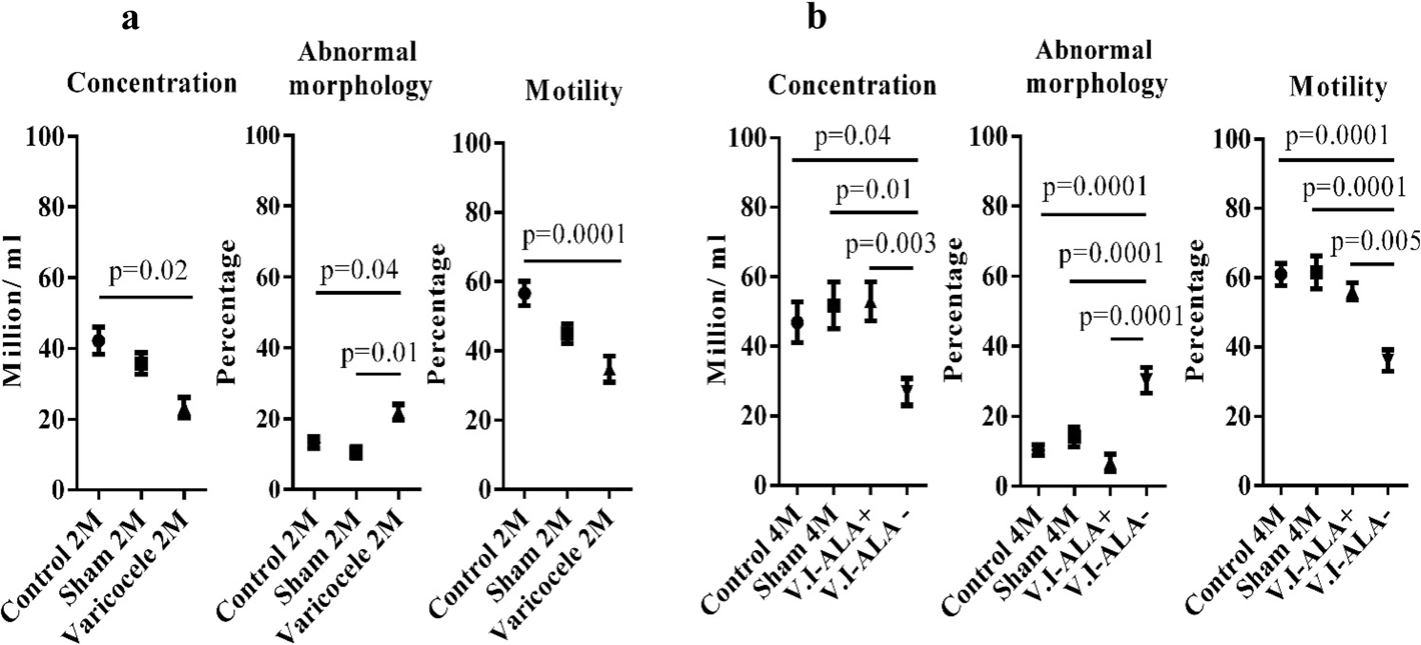
Comparison of sperm parameters. Legend: Comparison of sperm parameter between groups at 2 (**a**) and 4 (**b**) months post-surgery or induction of varicocele. Sperm concentration and motility decreased significantly after 2 M varicocele compared to the 2 M control, while the abnormal morphology showed a significant increase. At 4 months, sperm concentration and motility decreased in the V.I-ALA^−^ group, compared to the V.I-ALA^+^ group and the control group. Abnormal morphology increased significantly in the V.I-ALA^−^ group compared to the 4 M control group, the 4 M sham group and the V.I-ALA^+^ group. V.I-ALA^+^: varicocele rats treated with ALA for 2 to 4 months post induction of varicocele, V.I-ALA^−^: varicocele rats treated with water as solvent of ALA for 2 to 4 months post induction of varicocele. Statistical analysis (one way ANOVA) for each time point (2 or 4 M) and for each parameter was carried out between groups and *p*-value is provided for the groups which showed significant difference (All the data were presented as Mean ± SEM). M: month post-surgery or varicocele induction (V.I). V.I-ALA^+^: varicocele rats treated with alpha lipoic acid, V.I-ALA^−^: varicocele rats treated with water as solvent

As shown in Fig. 2a, the mean sperm concentration was significantly lower in VCL animals than in control animals at 2 months of age. Abnormal morphology was also significantly higher in the VCL group compared to control and sham animals. Sperm motility was also significantly lower in the group of VCL animals compared to the control group. The same observations apply to animals at 4 months of age (V.I-ALA^−^; Fig. 2b) with significantly lower sperm concentration, higher abnormal morphologies and lower motility compared to the control group and/or the sham animals. It is interesting to note that when ALA was administered to the animals (V.I-ALA^+^), a significant improvement of these 3 parameters was observed with a sperm concentration going up, abnormal morphology going down, and sperm motility going up*.*

### Morphometric and histological parameters

At 2 months of age the ipsilateral testicular volume of the VCL animals (1.01 ± 0.07 cm^3^) was significantly different (*p* = 0.01) to that of the control animals (1.43 ± 0.05 cm^3^). Regarding the other morphometric parameters monitored none were significantly different between the two groups of animals.

The mean mitotic index, spermatogenesis index and Johnson score were significantly decreased when comparing the VCL 2 months group to the control and sham groups at the same age (Table 2). Similarly, at 4 months, these same parameters were also significantly decreased when the VCL animals (V.I-ALA^−^) were compared to the control and sham groups. The addition of ALA (V.I-ALA^+^ group) significantly improves these parameters compared to VCL, although it did not bring them back to the level of the control animals (Table 2).

**Table 2:** Comparison of histological parameters and Johnson score between groups

Group	TD (μm)	MI (%)	SP (%)	JS
**Control** **(2 M)**	8.07 ± 0.02**a**	68.24 ± 0.39**d**	90.21 ± 0.52 **h**	9.41 ± 0.57 **l**
**Sham** **(2 M)**	7.80 ± 0.26	67.52 ± 0.09**e**	89.21 ± 0.45**i**	8.16 ± 0.75 **m**
**V.I (2 M)**	7.38 ± 0.04**a**	31.92 ± 0.78**de**	30.27 ± 0.93**hi**	6.04 ± 0.45 **lm**
**Control** **(4 M)**	7.97 ± 0.31**b**	67.35 ± 0.79**f**	90.04 ± 1.05**j**	9.84 ± 1.25**n**
**Sham** **(4 M)**	7.68 ± 0.17	67.08 ± 0.47 **g**	88.00 ± 0.57 **k**	9.00 ± 0.30**o**
**V.I-ALA**^−^	7.44 ± 0.16**bc**	29.88 ± 1.42 **fg**	32.27 ± 0.74**jk**	5.43 ± 0.58**no**
**V.I-ALA**^**+**^	8.10 ± 0.07**c**	57.92 ± 0.44 **fg**	67.56 ± 0.97**jk**	7.53 ± 0.15**no**

In the 2 M VCL group, measures of MI, SP and JS showed a significant decrease compared to the control and sham groups. While TD showed a significant decrease compared to the control but not sham group. At 4 months, measures of TD, MI, SP and JS were significantly decreased in the V.I-ALA^−^ group compared to the control and treated group (V.I-ALA^+^). *ALA* Alpha lipoic acid, *M* Month post-surgery or varicocele induction (V.I), *TD* Tubular diameter, *EH* Epithelium height, *MI* Mitotic index, *SP* Spermatogenesis percentage and *JS* Johnson score. Statistical analysis (one way ANOVA) for each time point (2 or 4 M) and for each parameter was carried out between groups and the groups which showed significant different (*p*-value < 0.05) were defined by common alphabetic letters

The mean diameter of the testicular tubules was slightly but significantly decreased in VCL animals compared to controls at 2 months of age but not against the sham group (Table 2). The situation was not worse at 4 months of age, as we only recorded a tendency to decrease. ALA supplementation restored testicular tubule diameter to that of the control animals (Table 2).

**Fig. 3:**
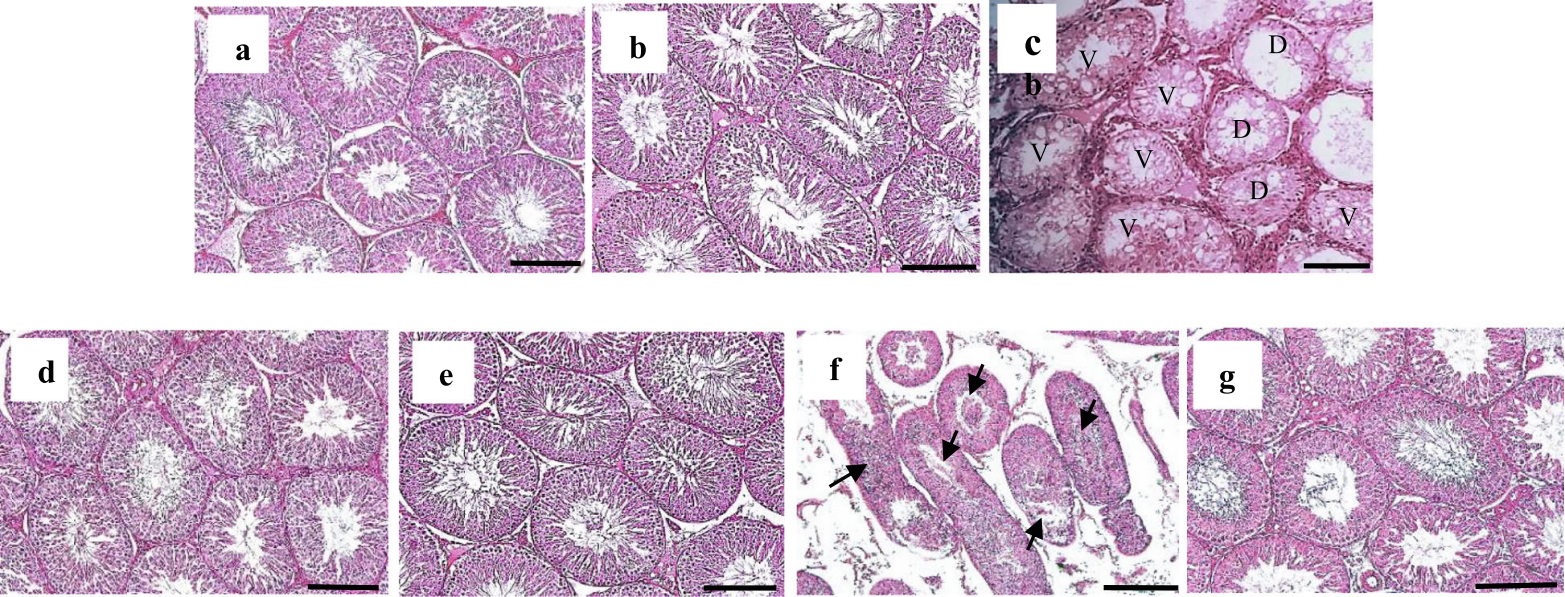
Testicular histological evaluation. Legend: Eosin and Hematoxilyn staining. **a** Control 2 M, **b** Sham 2 M, **c** Varicocele 2 M, **d** Control 4 M, **e**. Sham 4 M, **f**. V.I-ALA^−^ (varicocele rats treated with water as solvent of ALA for 2 to 4 months post induction of varicocele) and **g** V.I-ALA^+^ (varicocele rats treated with ALA for 2 to 4 months post induction of varicocele). Intact seminiferous tubules are seen in (**a**), **b** and **d** and **e** while vacuolated seminiferous epithelium (V) and disrupted spermatogenesis (D) are present in varicocele group (**c**) and distorted seminiferous tubules with immature germ cells present in the lumen (arrows) are present in V.I-ALA^−^ groups (**f**). ALA: Alpha lipoic acid, D: Disrupted spermatogenesis, M: month post-surgery or varicocele induction (V.I), V: Vacuolated seminiferous epithelium, V.I-ALA^+^: varicocele rats treated with alpha lipoic acid, V.I-ALA^−^: varicocele rats treated with water as solvent. Magnification bar (200 μm)

The histological evaluation of the testes by hematoxilin and eosin staining is presented separately (Fig. 3). As can be seen, the seminiferous tubules are intact in the control and sham group and in the 2 and 4 month groups (Fig. 3a, b and d, e), while the seminiferous epithelium appears vacuolar and spermatogenesis is disturbed in the varicocele group at 2 months (Fig. 3c). Similarly, deformed seminiferous tubules with immature germ cells present in the lumen are visible in the V.I-ALA^-^ group (Fig. 3f), whereas all these abnormalities were almost absent in the V.I-ALA^+^ group (Fig. 3g).

### Testicular Fe3+ distribution

Perl’s staining results revealed that the mean distribution of testicular Fe^3+^ was significantly higher in the VCL groups than in the control and sham groups whatever the age. The ALA treatment significantly reduced the testicular Fe^3+^ representation compared to the VCL group (Table 3).

**Table 3:** Comparison of percentage of Fe^3+^ distribution between groups

^**Fe3+ distribution (%)**^							
Group	Control(2 M)	Sham (2 M)	V.I (2 M)	Control (4 M)	Sham (4 M)	V.I-ALA ^**-**^	V.I-ALA^**+**^
**Mean ± SEM**	9.72 ± 0.24**a**	8.90 ± 0.12**b**	37.15 ± 0.57**ab**	9.56 ± 0.26**c**	9.48 ± 0.28**d**	52.65 ± 1.47 **cd**	14.96 ± 0.24 **cd**

There is a significant increase in Fe^3+^ accumulation in the 2 M varicocele group compared to the 2 M control and sham groups. Fe^3^^+^ accumulation decreased significantly in the V.I-ALA^+^ group compared to the V.I-ALA^−^, 4 M control and sham groups. *ALA* Alpha lipoic acid, M: month post-surgery or varicocele induction (V.I), V.I-ALA^+^: varicocele rats treated with ALA^+^ for 2 to 4 months post induction of varicocele, V.I-ALA^−^: varicocele rats treated with water as solvent of ALA for 2 to 4 months post induction of varicocele. Statistical analysis (one way ANOVA) for 4 M and for each parameter was carried out between groups and the groups which showed significant different (*p*-Value < 0.05) were defined by common alphabetic letters

### Sperm chromatin maturation, DNA fragmentation and lipid peroxidation

**Fig. 4:**
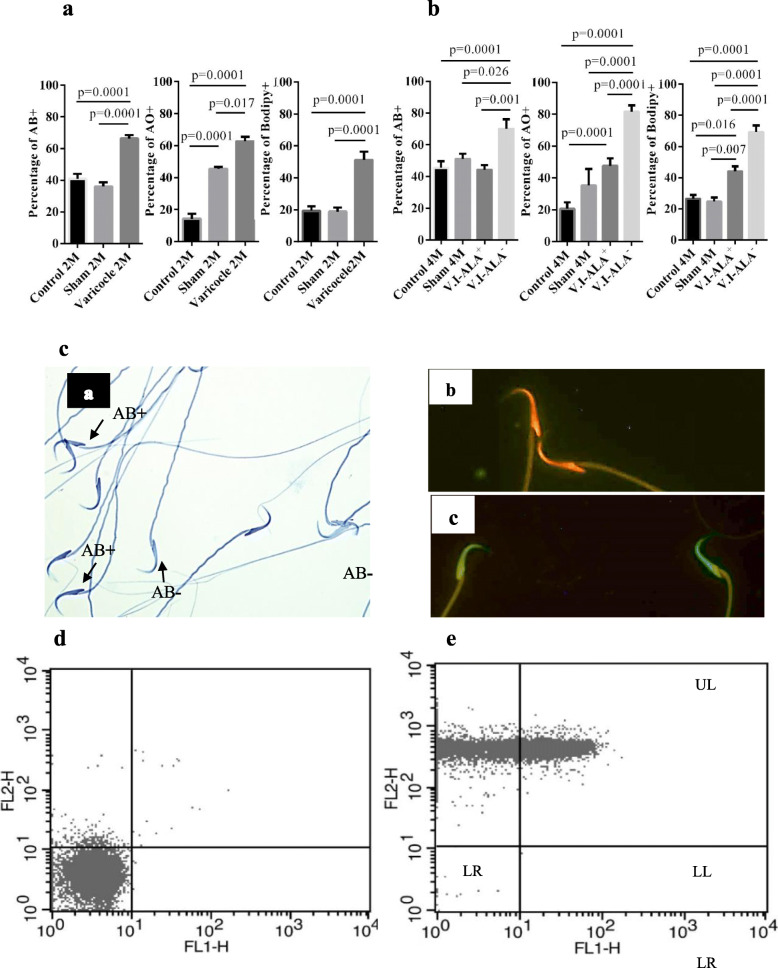
Evaluation of sperm functional tests. Legend: Aniline blue, Acridin orange and BODIPY staining at **a**) 2 month and **b**) 4 months post-surgery. Aniline blue, acridine orange and bodipy tests all indicated a significant increase in sperm alterations at 2 M varicocele compared to the 2 M control and sham groups. At 4 months of VCL, the percentage of aniline blue, acridine orange and bodipy positive spermatozoa were significantly higher in the V.I-ALA^−^ group compared to the treated group (V.I-ALA^+^) and the 4 M control and sham groups. Statistical analysis (one way ANOVA) for each time point (2 or 4 M) and for each parameter was carried out between groups and *p*-value is provided for the groups which showed significant difference. **c**) a) aniline blue staining, sperm heads with dark blue staining indicate anilin blue positive and light blue staining indicates anilin blue negative. b) acridin orange positive sperm heads show orange-red fluorescence light indicate damaged chromatin, c) acridin orange negative sperm heads that expose green fluorescence light indicate intact chromatin. D) Evaluation of lipid peroxidation by bodipy C11 staining, 2-D flow cytometry dot plot of fluorescence intensity, for each sample, approximately 10,000 sperm cells were counted. **d**) shows unstained sperms while **e**) shows stained ones. The upper right quadrant, represents C11 Bodipy positive spermatozoa at 530 nm in green fluorescence with a FL1 band pass (BP).AB+: anilin blue positive, AB-: anilin blue negative, AO+: acridin orange positive, ALA: Alpha lipoic acid, Bodipy +: Bodipy positive, LL: Lower left, LR: lower right, UL: Upper left, UR: upper right, M: month post-surgery or varicocele induction (V.I), V.I-ALA^+^; varicocele rats treated with ALA for 2 to 4 month post induction of varicocele, V.I-ALA^−^: varicocele rats treated with water as solvent of ALA for 2 to 4 month post induction of varicocele. Magnification bar (50 μm)

As shown in Fig. 4A and B, the percentages of aniline blue (AB) positive spermatozoa in VCL animals at either 2 or 4 months of age were significantly higher than in control and sham animals at the same ages. ALA supplementation significantly reduced the percentage of AB positive spermatozoa to the level of control animals.

With regard to DNA fragmentation (Fig. 4A and B), the percentages of acridine orange (AO)-positive sperm were also significantly higher in the VCL groups compared to the control and sham animals regardless of age. Here again, the ALA treatment significantly reduced the percentage of AO-positive sperm but failed to bring it back to the level of the control animals.

Similarly, evaluation of lipid peroxidation of sperm membranes by the Bodipy C11 probe showed that mean percentages of positive spermatozoa were significantly higher under VCL conditions compared to control and sham animals at all ages. Treatment with ALA significantly reduced the percentage of positive spermatozoa but again, it did not reach the level of the control animals (Fig. 4C). The aniline blue and acridine orange stains are shown in Fig. 4C (a and b, c, respectively) while Figs. 4D and E show the bodipy C11 charts.

### Relative expression of ferroptosis markers P53, Nrf2 and Slc7a11

**Fig. 5:**
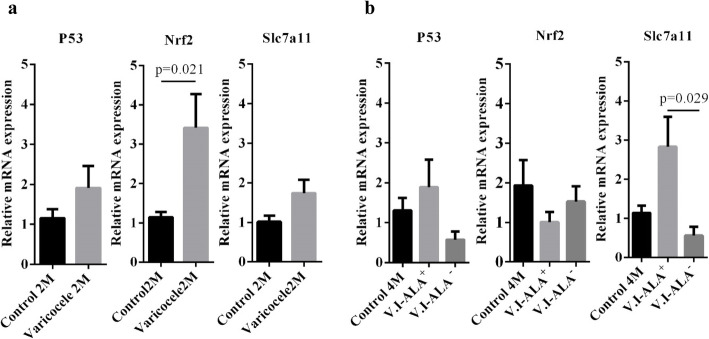
Comparison of relative mRNA expression. Legend: Comparison of relative expression of *P53, Nrf2* and *Slc7a11* mRNA in different groups at 2 (**a**) and 4 months (**b**) post induction varicocele. The relative expression of Nrf2 showed a significant increase in the varicocele group compared to the 2 M control group. At 4 months, the relative expression of *Slc7a11* was significantly increased in the treated group (V.I-ALA^+^) compared to the 4 M varicocele group (V.I-ALA^−^). M: month post-surgery or varicocele induction**,** Nrf2: Nuclear factor erythroid 2–related factor 2, V.I-ALA^+^: varicocele rats treated with ALA for 2 to 4 months post induction of varicocele, V.I-ALA^−^: varicocele rats treated with water as solvent of ALA for 2 to 4 months post induction of varicocele. Statistical analyses (independent sample T-test) for 2 months, (one way ANOVA) for 4 M samples, for each parameter were carried out between groups and *p*-value is provided for the groups which showed significant difference

Figure 5a shows that although the three monitored ferroptosis markers tended to accumulate in greater proportion in 2 months old VCL animals compared to control animals, only the accumulation of *Nrf2* mRNA was significantly different. Intriguingly, in VCL animals at 4 months of age *Nrf2* mRNA accumulation was not different to that of control animals (Fig. 5b). At this age, it is also intriguing that the accumulation of *Slc7a11* mRNA decreased in VCL animals compared to controls and, that ALA supplementation resulted in a significant increase in this mRNA steady state level (Fig. 5b).

**Fig. 6:**
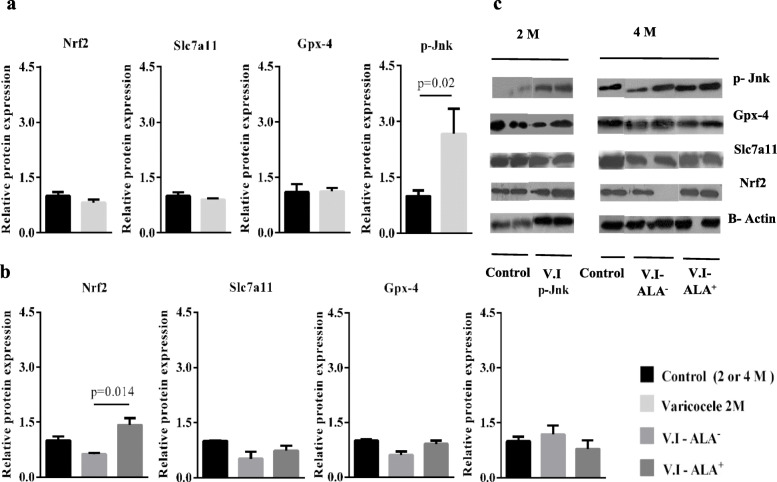
Relative protein expression between groups. Legend: **a** Relative protein expression of Nrf2, Slc7a11, Gpx-4 and p-Jnk between groups at 2 months. **b** Relative protein expression of Nrf2, Slc7a11, Gpx-4 and p-Jnk between groups at 4 months. **c** Western blot bands representative for Nrf2, Slc7a11, Gpx-4, p-Jnk and ß-actin as housekeeping protein in 2 and 4 months groups. A minimum of 3 replicates were carried out. Evaluation of the relative expression of Nrf2 showed a significant increase in the V.I-ALA^+^ group compared to the V.I-ALA^−^ group. Evaluation of the relative expression of p-Jnk showed a significant increase in the 2 M varicocele group compared to the 2 M control group. ALA: Alpha lipoic acid, Gpx-4: glutathione peroxidase-4, M: month post-surgery or varicocele induction (V.I), Nrf2: nuclear factor erythroid 2–related factor, p-Jnk: phosphorylated c-Jun N-terminal kinase, V.I-ALA^+^: varicocele rats treated with ALA^+^ for 2 to 4 months post induction of varicocele, V.I-ALA^−^: varicocele rats treated with water as solvent of ALA for 2 to 4 months post induction of varicocele. All data are presented as Mean ± SEM. p-value is provided for the groups which showed significant difference

Since, beside transcriptional activation, signaling cascades may rely on post-transcriptional responses leading to higher level of proteins we have monitored the Nrf2 and Slc7a11 protein contents in control and VCL animals. Reflecting the weak transcriptional up-regulation, we found that the Nrf2 and Slc7a11 protein content of VCL animals were not that different to that of control animals whatever their age (2 months or 4 months; Fig. 6a, b). Interestingly, at 4 month of age, ALA supplementation resulted in a higher level of Nrf2 protein content (which was statistically significant only when compared to V.I-ALA^−^ animals but not to control animals; Fig. 6b). No differences were recorded in any conditions for Slc7a11 (Fig. 6a, b). Western blot bands are presented in Fig. 6c.

### Relative expression of p-Jnk and Gpx-4 proteins

Since JNK and Gpx-4 are known targets for the markers of ferroptosis evaluated above, we monitored the activation of Jnk by its level of phosphorylation (p-Jnk) and the accumulation of the Gpx-4 protein (a target of Nrf2). We found that the p-Jnk content of the testes was significantly higher in the VCL group than in the control animals at 2 months of age. At the same age, we did not observe any variation in the representation of the Gpx-4 protein. At 4 months of age, neither p-Jnk nor Gpx-4 was significantly more represented in the VCL testes than in the testes of control animals. ALA supplementation did not change anything for these two targets in the VCL animals.

### GSH and NADPH contents

**Fig. 7:**
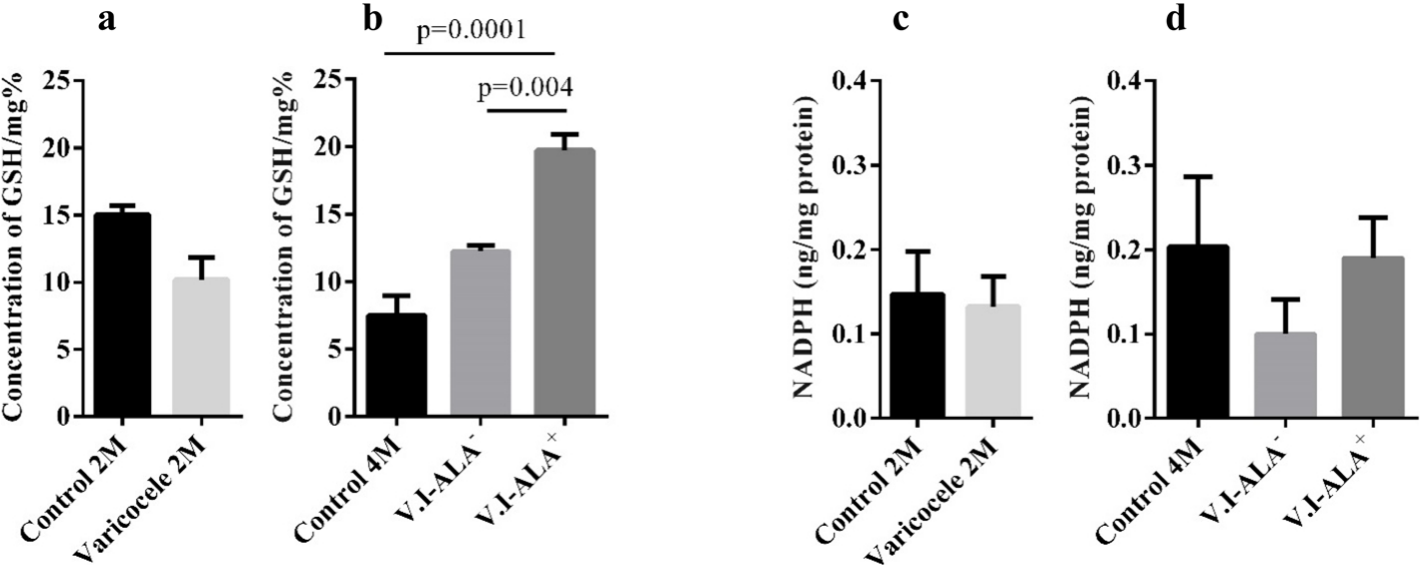
GSH and NADPH content in the different groups of animals. Legend: Evaluation of GSH content in **a** 2 M group and **b** 4 M group. Evaluation of NADPH content in **c** 2 M group and **d** 4 M group. Evaluation of the GSH concentration showed a significant increase in the V.I-ALA^+^ group compared to the V.I-ALA^−^ group and the 4 M control group. ALA: Alpha lipoic acid, GSH: Glutathione, M: month post-surgery or varicocele induction (V.I), NADPH: Reduced nicotinamide adenine dinucleotide phosphate, V.I-ALA^+^: varicocele rats treated with ALA^+^ for 2 to 4 months post induction of varicocele, V.I-ALA^−^: varicocele rats treated with water as solvent of ALA for 2 to 4 months post induction of varicocele. All data are presented as Mean ± SEM. p-value is provided for the groups which showed significant difference

As shown in Fig. 7, GSH and NADPH levels were not significantly different between the VCL (10.20 ± 1.67, 0.13 ± 0.03) and control (14.36 ± 0.18, 0.14 ± 0.05) groups at 2 months, while at 4 months GSH levels increased significantly in the V.I-ALA^+^ group (19.75 ± 1.18, 0.12 ± 0.04) compared to the control group (7.50 ± 1.44; *p* = 0.0001) and the VCL ALA^−^ group (12.25 ± 0.47; *p* = 0.004).

## Discussion

Despite the clearly established link between VCL and male infertility, VCL has remained a subject of debate for more than half a century. This is in part due to an incomplete understanding of the molecular mechanisms at stake. Among the underlying molecular mechanisms, the high level of ROS that accompanies VCL appears to play a key role in its etiology [9, 21]. ROS can initiate different cell death pathways such as apoptosis, necrosis and also ferroptosis [22, 23]. This present study aimed to show whether ferroptosis is involved in VCL in particular in the light that iron was shown to accumulate within the VCL testis [9].

We first confirmed in a well-established and controlled rat VCL model that we found all the sperm characteristics of VCL including^:^ reduction in classical spermatic parameters (mean concentration, normal morphology, motility), increased loss of spermatic chromatin integrity (assessed by the higher presence of residual histones), and increased lipid peroxidation and DNA damage 2 months after surgery-induced VCL These characteristics are largely attributable to oxidative stress generated by the VCL situation [12, 24–26]. In addition, we also confirm that the iron content was significantly increased in the testes of VCL animals (by 5-fold and by 7-fold respectively in 2 and 4 months old animals) compared to the control group. This situation, where both ROS and iron are in excess, makes it quite possible to induce ferroptosis in the VCL testis.

As stated in the introduction, the molecular pathway of ferroptosis is relatively distinct from that of the apoptosis pathway. In particular, p-Jnk, indirectly by regulating p53 transcription and activity, plays a key role in the ferroptosis pathway by inhibiting the Nrf2-mediated antioxidant response. However, despite the fact that Jnk kinase activity seems necessary for ferroptosis, its exact involvement has yet to be elucidated [27–29]. In this context, as understood to date, a decrease in the expression of Nrf2 target genes such as *Slc7a11* and *Gpx-4* should be expected [30, 31]. We show here that, in agreement with the above scenario, the activated protein p-Jnk was significantly more represented in the VCL testis group at 2 months of age. Regarding Nrf2, *Nrf2* mRNA accumulation was found to be higher in the testis of 2 months-old VCL animals when compared to control testis at the same age but this was not followed by an elevated level of Nrf2 protein. Concurring with the unchanged level of Nrf2 transcriptional activator of antioxidant genes, we show that one of its well-known target gene, *Gpx-4*, is not activated in the VCL testis (as the GPx4 content of the VCL testis did not change neither in 2 months nor in 4 months groups). Furthermore, since neither GSH nor NADPH levels were modified in the 2 months nor 4 months Varicocele-testis this allows us to suggest that the classical antioxidant response is not significantly activated as GSH and NADPH are the co-factors necessary for the recycling of the Gpx enzymatic family in particular. In addition, following the assumed scenario, one would expect p-Jnk increased level to result in diminished expression of *Slc7a11.* In contrast to what was expected, we observed an increased accumulation of *Slc7a11* mRNA in the 2 months VCL testis when compared to control testis at the same age.

Taken together, these data strongly suggest that despite intrinsic characteristics that would favors the induction of ferroptosis (i.e., high iron content, oxidative stress), ferroptosis is not active in the VCL testis. However, one should be cautious with such a statement as it is known that in the VCL, the testis is not homogeneously affected by the stress response. While some testicular regions are severely affected to the point that it leads to spermatogenesis arrest and tubular destructuring, other parts of the VCL testis are much less damaged and still support spermatogenesis, although under stress [32]. In this mixed context, it is possible that the molecular changes that occur in areas of severe VCL damage are levelled out by areas of mild damage. This mixed context in terms of severity may also explain why some people with VCL are fertile while others are infertile.

Considering the potential beneficial effect of ALA supplementation on the VCL testis, we observed that ALA significantly improved sperm parameters and Johnson’s score. ALA also reduced the loss of sperm nuclear integrity, resulting in better nuclear condensation and a lower level of DNA fragmentation. Finally, ALA also helped reduce sperm membrane lipid peroxidation. Overall, these beneficial effects of ALA on the VCL rat testis argue for its use as adjunctive therapy in VCL treatment options. This is consistent with our recent clinical trial showing that ALA supplementation after varicocelectomy improves sperm parameters [33]. ALA had no significant effect on Jnk phosphorylation, although it tended to be slightly reduced. However, ALA increased the Nrf2 content of the testis, which most likely explains the observed increase in Slc7a11 and Gpx-4 protein levels in the testes of ALA supplemented VCL rats. Similarly, ALA supplementation resulted in an increase in testicular GSH levels. Taken together, these effects are consistent with the known beneficial impact of ALA on Nrf2-mediated antioxidant response [34]. Some reports have already suggested that Nrf2 may act as an anti-ferroptotic protein by stimulating the antioxidant response [7, 35]. Indeed, a recent study showed that MPP (1-methyl-4-phenylpyridinium: Parkinson’s disease inducer) in PC12 cells induce oxidation and ferroptosis and that ALA was capable of reversing oxidation and ferroptosis markers [36]. Thus, we can hypothesize that the sensitive Nrf2 testicular pathway may function as a blocker of ferroptosis in V.I-ALA^+^, by further inducing the testicular Nrf2 pathway, may be a way to further protect the testis from Varicocele-mediated damage. In addition to its reinforcing effect on the Nrf2-mediated antioxidant testicular response, ALA may also have other beneficial effects on the VCL testis, helping it to stay away from ferroptosis. Indeed, ALA is known to function as a chelator for transition metals such as Fe and Cu [37, 38]. This may well explain why we observed a reduced level of Fe^3+^ in ALA supplemented VCL rats (at 4 months of age compared to non-supplemented rats). In addition, ALA has been reported to protect enzymatic and non-enzymatic antioxidants, including coenzyme Q10, GSSG, vitamins C and E [39], to capture ROS and nitrogen species, and to act as a cofactor for enzymes, particularly those required for the Krebs cycle [40, 41]. Overall, ALA may help, in the case of the VCL testis, to preserve spermatozoa from oxidative damage that is closely related to motility and viability losses.

## Conclusion

The data presented here are not in support of a clear ferroptosis situation in the rat VCL testis. It is important to note that this could be due to the heterogeneous context of the VCL testis showing regional damages eventually masking the detection of the ferroptosis classical molecular markers. It may be possible to clarify the situation in the future through tissue laser capture experiments to differentiate the presence of ferroptose markers in intact and damaged seminiferous tubules. We also show here that ALA supplementation in situation of VCL could be a pertinent option as ALA was shown to stimulate the Nrf2-mediated testis antioxidant response leading to improved spermatozoa structural and functional parameters. Whether an iron chelator such as Deferasirox (DFX), in addition to ALA supplementation, could benefit patients with VCL will have to await further validation.

## Data Availability

The datasets used and/or analyzed during the current study are available from the corresponding author on reasonable request.

## Abbreviations

AB: Aniline blue
AO: Acridin orange
ALA: Alpha-lipoic acid
bp: Base pair
°C: Degrees Celsius
D: Disrupted spermatogenesis
DFX: Deferasirox
EH: Epithelium height
GSH: Glutathione
GPX-4: Gluthation peroxidase-4
JS: Johnson score
LL: Lower left
LR: Lower right
UL: Upper left
UR: Upper right
M: Month post-surgery or varicocele induction
MI: Mitotic index
MPP: 1-methyl-4-phenylpyridinium
n: Number of rats
NADPH: Reduced nicotinamide adenine dinucleotide phosphate
NRF2: Nuclear factor erythroid 2–related factor 2
p-JNK: Phosphorylated c-Jun N-terminal kinase
SP: Spermatogenesis percentage
TD: Tubular diameter
V: Vacuolated seminiferous epithelium
VCL: Varicocele
V.I: Varicocele induction
V.I-ALA^+^: Varicocele rats treated with ALA for 2 to 4-month post induction of varicocele
V.I-ALA^−^: Varicocele rats treated with water as solvent of ALA for 2 to 4-month post induction of varicocele
